# The southern ocean meridional overturning in the sea-ice sector is driven by freshwater fluxes

**DOI:** 10.1038/s41467-018-04101-2

**Published:** 2018-05-03

**Authors:** Violaine Pellichero, Jean-Baptiste Sallée, Christopher C. Chapman, Stephanie M. Downes

**Affiliations:** 1 Sorbonne Universités, UPMC Univ., Paris 06, UMR 7159, LOCEAN-IPSL, F-75005 Paris, France; 2 Antarctic Climate and Ecosystems Cooperative Research Centre, University of Tasmania, Hobart, 7001 Australia

## Abstract

The oceans are traversed by a large-scale overturning circulation, essential for the climate system as it sets the rate at which the deep ocean interacts with the atmosphere. The main region where deep waters reach the surface is in the Southern Ocean, where they are transformed by interactions with the atmosphere and sea-ice. Here, we present an observation-based estimate of the rate of overturning sustained by surface buoyancy fluxes in the Southern Ocean sea-ice sector. In this region, the seasonal growth and melt of sea-ice dominate water-mass transformations. Both sea-ice freezing and melting act as a pump, removing freshwater from high latitudes and transporting it to lower latitudes, driving a large-scale circulation that upwells 27 ± 7 Sv of deep water to the surface. The upwelled water is then transformed into 22 ± 4 Sv of lighter water and 5 ± 5 Sv into denser layers that feed an upper and lower overturning cell, respectively.

## Abstract (web summary)

The Southern Ocean is key in sustaining the world’s ocean global circulation, yet the influence of its freshwater cycle remains unconstrained. Here, the authors use a detailed oceanographic database to evaluate surface buoyancy fluxes and reveal central roles for both precipitation and sea-ice formation and melt.

## Introduction

**Fig. 1:**
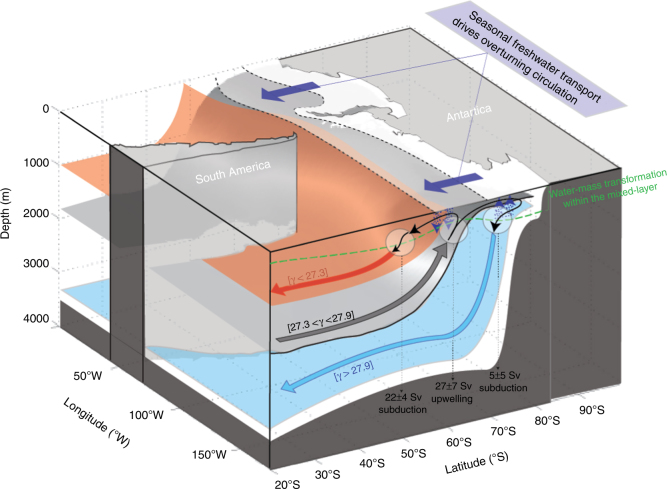
Water-mass transformation within the Southern Ocean mixed-layer under sea-ice. Schematic cross-section illustrating the main water-masses in the Southern Ocean (Antarctic Intermediate and Mode Waters in red, Circumpolar Deep Waters in gray, and Dense Shelf Waters and Antarctic Bottom Waters in blue) and their interaction with ice and the surface. The water-masses are denoted by their neutral density values and the arrows corresponding to each water-masses indicate subduction (downward) or upwelling (upwards). The violet arrows illustrate the effect of northward sea-ice extent and freshwater transport. The green line is the mixed-layer

The Southern Ocean is a known region of intense water-mass transformation and formation^1,2^ and thereby plays a central role in the climate system by controlling the rate at which ocean reservoirs of tracers, such as heat and carbon, communicate with the surface. The upper cell of the meridional circulation in the Southern Ocean (Fig. 1) is commonly recognized as a wind-driven circulation, with Circumpolar Deep Water (CDW) upwelling along steeply tilted isopycnals, driven by divergent Ekman transport at the surface. CDW originates from a range of deep waters that enter into the Southern Ocean, and upwells in the Antarctic Circumpolar Current (ACC)^3,4^. At the surface CDW is exposed to surface buoyancy fluxes, then transforms into lighter Subantarctic Mode Water (SAMW) and Antarctic Intermediate Water (AAIW); these water-masses constitute the upper branch of the Meridional Overturning Circulation^5^. The Southern Ocean is also a major source of dense Antarctic Bottom Water (AABW), which forms primarily in the Ross and Weddell Seas, and along the eastern coast of the Antarctic continent^6–8^. In these regions, the intense surface buoyancy fluxes associated with the interactions between the ocean and the atmosphere, ice shelves, and sea-ice, produce cold and salty Dense Shelf Water (DSW), which is transformed into AABW that fills most of the world’s oceans^9–11^.

Recently, the Southern Ocean freshwater cycle, of which sea-ice formation and melting is an important component, has been suggested to play an important role in driving the large-scale overturning circulation^12^. However, due to the logistical challenges in acquiring direct observations of atmosphere-ice-ocean fluxes in remote regions and under sea-ice, the link between the large-scale ocean circulation and freshwater cycle have only been estimated using data assimilating numerical models, in which fluxes and small scale physics are adjusted to best-match ocean observations, but remain questionable^12^.

In this paper, we present a novel and complementary analysis in which we estimate surface buoyancy fluxes and their impacts on ocean surface water-masses, without the use of a complex numerical model, but rather directly from an unprecedented database of ocean measurements under sea-ice; combining observations from ships, autonomous floats, and animal-born sensors^13^. Surface buoyancy fluxes received by the ocean surface mixed-layer from atmosphere, ice, and diapycnal mixing are inferred as a residual of observation-based mixed-layer heat and salt budgets (see Methods section, Eqs. (4) and (5)), and water-mass characteristics are derived from hydrographic observations^13^.

## Results

### Atmospheric and sea-ice buoyancy fluxes

**Fig. 2:**
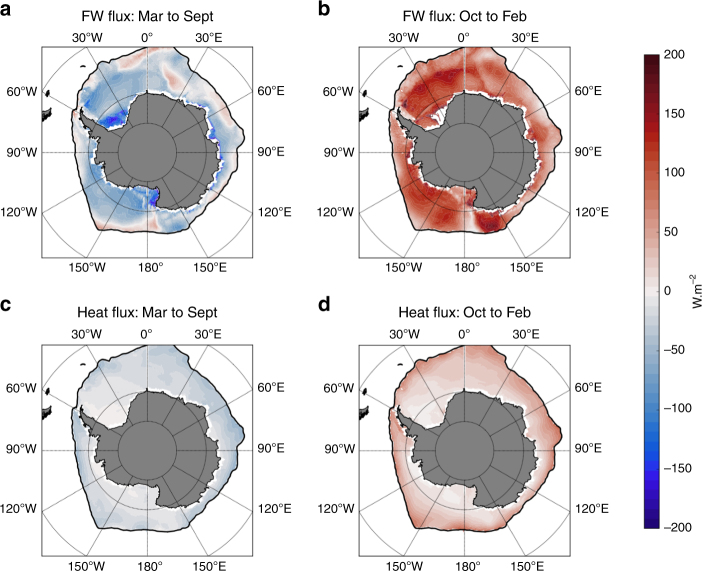
Surface buoyancy fluxes in the sea-ice sector. Winter (**a**,**c**) and summer (**b**,**d**) surface buoyancy fluxes derived from an observation-based mixed-layer buoyancy budget, and decomposed into surface freshwater flux (**a**,**b**) and surface heat flux (**c**,**d**). Units are equivalent W m^−2^. The positive values indicate a buoyancy gain into the mixed layer, and the negative values indicate a buoyancy loss into the mixed layer

The seasonal variation of the estimated net buoyancy fluxes to and from the surface mixed-layer in the sea-ice sector is shown in Fig. 2, with an overall loss of buoyancy in winter (Fig. 2a, c), and gain of buoyancy in summer (Fig. 2b, d) in the order of 100–200 W m^−2^ (in this paper, all buoyancy flux units are converted into an equivalent heat flux). Interestingly, it is the freshwater contribution that dominates the seasonal variation of the net buoyancy flux. The heat flux contributes only marginally (a factor ~2–5 lower than the freshwater contribution), and mostly in regions near the winter sea-ice edge that spend much of the year ice-free. The relative contribution of freshwater and heat to the net buoyancy flux is consistent with sea-ice partially isolating the ocean from atmospheric heat fluxes, combined with large freshwater fluxes associated with brine rejection and ice melt. Additionally at near freezing temperatures, the thermal expansion coefficient of sea-water is close to zero^14^, meaning the density of water is quite insensitive to heat fluxes.

The geographical pattern of buoyancy fluxes presents a large-scale meridional gradient, with the largest fluxes near the Antarctic coast. This meridional gradient is largely dominated by the freshwater flux contribution (Fig. 2), and is more marked in winter than in summer. Even though we are unable to disentangle from our dataset the contribution of sea-ice, precipitation, and diapycnal mixing, sea-ice is likely a strong contributor to the freshwater flux^13^. The meridional gradient in the buoyancy flux and its seasonal evolution is consistent with the fact that the largest amount of ice formation occurs near the Antarctic coast on continental shelves, while ice melt tends to be more spread out after ice has been exported away from its formation region by wind and currents^15^.

### Water-mass transformation and related vertical circulation

**Fig. 3:**
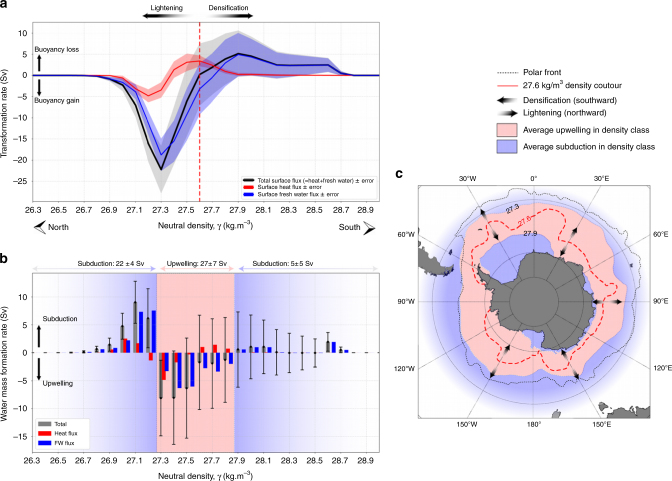
Components of water-mass transformation and formation and the location of subduction and upwelling regions. **a** The annual-mean water-mass transformation (Sv) in the sea-ice sector in neutral density coordinates. The total transformation (black line) is a combination of the surface atmosphere-ocean-ice freshwater flux (blue line) and heat flux (red line). Shaded areas correspond to the estimated errors for the total surface buoyancy flux (gray), the surface freshwater flux (blue), and surface heat flux (red); see Methods section for details on the estimate of error. The vertical red line corresponds to the 27.6*γ*, which is found to mark the division between water that becomes lighter and water that becomes denser. **b** The annual-mean water-mass formation rate in Sv integrated in 0.1 kg m^−3^ density bins (gray bars), divided into the freshwater (blue bars) and heat (red bars) contributions. The error bars are propagated from errors shown in panel **a**. **c** Observed outcrop of isopycnal 27.3*γ*, 27.6*γ*, and 27.9*γ*, that denote the subduction regions (blue areas) and upwelling regions (red areas) as in panel **b**. Dotted black lines shows the mean position of the Polar Front^23^

In 1982, Walin^16^ proposed a framework to estimate the annual-mean water-mass transformation from knowledge of surface heat fluxes. Using this framework and the net mixed-layer buoyancy fluxes presented above, we derive the annual-mean water-mass transformation in the mixed layer in the Antarctic sea-ice zone (See Methods section; Eq. (2); Fig. 3a). These water-mass transformation rates show buoyancy gain in the lightest density class encountered in the sea-ice sector (*γ* ≤ 27.6 kg m^−3^), and loss of buoyancy in the heaviest density class (*γ* ≥ 27.6 kg m^−3^). The buoyancy gain peaks at 27.3*γ*, reaching ~ −22 ± 4 Sv of transformation directed toward lighter waters, while the loss of buoyancy peaks at 27.9*γ*, yielding ~ 5 ± 5 Sv of transformation toward heavier waters (for details on error estimates, see Methods section).

The net water-mass transformation has a strong seasonal cycle (Supplementary Note 1). In fall and winter, water-masses are transformed into denser waters, while in spring and summer water-masses are transformed into lighter waters, but act within lighter density classes (Supplementary Note 1; Supplementary Fig. 1). Such a seasonal cycle is consistent with the influence of melting and refreezing sea-ice, as brine rejection associated with ice formation in fall/winter would densify the dense water-masses that lie close to the Antarctic continent where sea-ice forms. Lightening in spring and summer is instead more spread out in density, and shifted to lighter density classes consistent both with the seasonal cycle of the upper ocean and with the northward ice advection toward lighter density class areas after its formation. As an attempt to quantify the impact of sea-ice in our derived water-mass transformation, we use a recent estimate of ice–ocean fluxes^17^ from which we derive an independent water-mass transformation. In addition, we also estimate transformation by precipitation^18^ and iceberg melt^19^ (Supplementary Note 2; Supplementary Fig. 2). Although each freshwater flux product has numerous limitations (especially the precipitation flux; see Supplementary Note 2), they permit us to compare the relative order of magnitude of water-mass transformation by each component of the freshwater cycle within our estimate of the total buoyancy flux. The water-mass transformation rates obtained from the sum of the three products and those from our in situ observation-based estimates compare very well, suggesting that precipitation and sea-ice fluxes are the dominant contributors to water-mass transformation. This relatively good comparison gives us confidence in our water-mass transformation estimate. To further test the robustness of our calculations, we have compared our estimates of the total buoyancy flux with four other products (see Supplementary Note 3; Supplementary Fig. 4). Although there are differences in the details in the buoyancy flux distributions between the different products, all agree on the order of magnitude of the heat and freshwater fluxes, and their large-scale structure (See Methods section).

In the water-mass transformation framework, water-masses can either accumulate or reduce in volume in a given density class, which (in steady state) must be balanced by, subduction or upwelling, respectively, through the base of the ocean mixed-layer^16,20^ (Eq. (2)). The water-masses lighter than 27.3*γ* (Fig. 3b) subduct into the Southern Ocean interior, with a peak subduction at 9 ± 4 Sv in the density class 27.1 ± 0.05*γ* that is dominated by freshwater-driven water-mass transformation. This density range (27–27.2*γ*) corresponds to the dense mode waters and intermediate waters^5^ that constitute the upper branch of the Southern Ocean meridional overturning circulation (Fig. 1, red arrow). Upper Circumpolar Deep Waters (27.3–27.8*γ*; Fig. 1, gray arrow) primarily upwells into the mixed-layer from the ocean interior. The errors are large when computing subduction/upwelling on narrow density bins (0.1*γ*; Fig. 3b), but the signal-to-noise ratio improves when computing subduction/upwelling over wider density range (26.3–27.3*γ*; 27.3–27.9*γ*; 27.9–28.8*γ*; Fig. 3b). The overall net upwelling is 27 ± 7 Sv, distributed relatively evenly over the entire 27.3–27.9*γ* density range, but slightly greater in the lighter part of the layer, with a peak upwelling of ~8 ± 8 Sv in each of the layer 27.3 ± 0.05*γ* and 27.4 ± 0.05*γ*. Water denser than ~ 27.8*γ* tends to subduct below the ocean surface (Fig. 1, blue arrow). Subduction peaks in the Lower Circumpolar Deep Waters density class (27.9–28.2*γ*), with smaller subduction rates also observed in the Dense Shelf Waters density class, 28.5–28.8*γ*. Overall, the subduction of dense Circumpolar Deep Waters and Dense Shelf Waters are the precursor to the formation of Antarctic Bottom Waters.

In agreement with previous studies based on large-scale inversion of the global ocean circulation^4,21,22^, we find that the density surface 27.3*γ* marks the approximate division between water that upwells and is converted into lighter waters, and water that upwells and is converted into denser waters (Fig. 3c). The upwelling density class, 27.3–27.9*γ*, outcrops circumpolarly in winter in large portions of the sea-ice sector. Density layers lighter than 27.3*γ* overall subduct and outcrop in winter close to the position of the polar front, consistent with a subduction of mode and intermediate waters in the vicinity of the Antarctic Circumpolar Current fronts^23^ (Fig. 3c). Water denser than 27.9*γ* also primarily subducts, but outcrops in winter close to the Antarctic continent in East Antarctica, and in the Ross and Weddell Seas.

### Transformation at regional scales

**Fig. 4:**
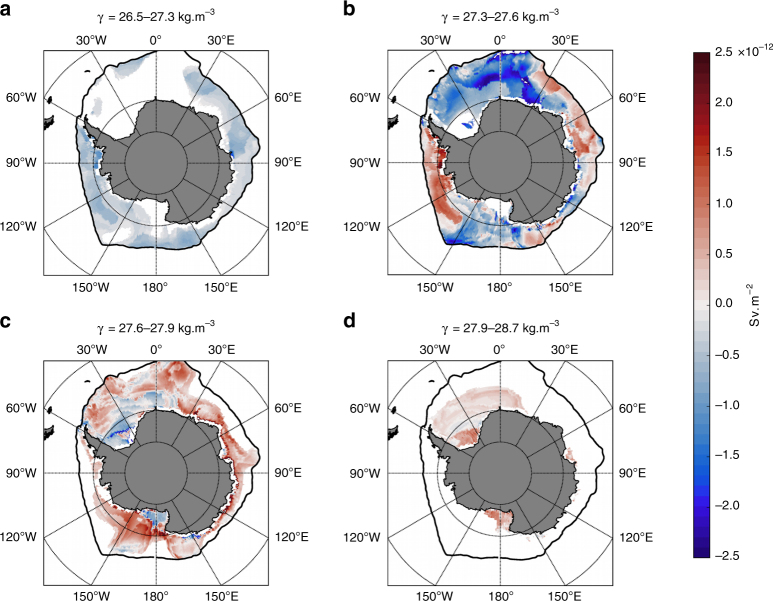
Regional distribution of the water-mass transformation in the sea-ice sector. Transformation (10^−12^ Sv m^−2^) across the isopycnals **a** 26.5–27.3, **b** 27.3–27.6, **c** 27.6–27.9, and **d** 27.9–28.7. The positive values of the water-mass transformation rate indicate a transformation toward higher density classes (buoyancy loss), and the negative values indicate a transformation directed toward lighter density classes (buoyancy gain)

The sign and magnitude of the annual-mean transformation rate varies regionally in each density classes (Fig. 4; see Brambilla et al.^24^ and Maze et al.^25^ for detailed discussion of the calculation of transformation maps). The transformation in the lightest layer 26.5–27.3*γ* is associated with a lightening that covers mostly the outer edge of the sea-ice sector (Fig. 4a). All sectors of the associated outcrop are consistently associated with an annual-mean lightening of the water-masses. In contrast, the annual-mean transformation in the Circumpolar Deep Waters density class (27.3–27.6*γ* and 27.6–27.9*γ*) varies in sign from one region to another. We find a dominant lightening of the lightest Circumpolar Deep Waters density layer, 27.3–27.6*γ*, slightly counterbalanced by densification (Fig. 4b) in primarily the Bellingshausen and Amundsen Seas. In denser Circumpolar Deep Waters, 27.6–27.9*γ*, the annual-mean transformation is almost exclusively densification in all regions, except the southern Weddell and Ross Seas (Fig. 4c). In particular, large densification of the layer 27.6–27.9*γ* occurs along the east-Antarctica continental shelf. Such large densification in this region must contribute to feeding the production of dense waters that ultimately form dense bottom waters^7,8^. Finally, the densest waters, in the range 27.9–28.7*γ*, are only observed in the well-known regions of dense water formation: the Weddell Sea, the Ross Sea, and the Prydz Bay (Fig. 4d). All of these regions are associated with densification of the densest Circumpolar Deep Waters into precursors of Antarctic Bottom Waters.

Annual-mean densification of waters adjacent to the Antarctic continental shelf is likely associated with the local convergence of water that would feed the bottom cell subduction. While a detailed local buoyancy budget (probably beyond what can be accomplished with the current observations system, given the associated errors) would be needed to further investigate this question, our analysis suggest that local convergence along the Antarctic coast occurs in different density classes in different regions: ~ 27.3–27.6*γ* in the Bellingshausen and Amundsen seas; ~ 27.6–27.9*γ* in East Antarctica; ~ 27.9–28.7*γ* in the Weddell and the Ross Seas, and the Prydz Bay. We note, however, that the local water-mass transformation associated with coastal processes and polynyas, while potentially key for transformation in the densest waters, are unlikely to be well represented in our observations.

In waters denser than 27.9*γ* in the Weddell sector, closed density contours allow estimation of the net subduction. We find that 4 Sv subducts in the Weddell sector, only slightly lower than the estimate of 6 ± 2 Sv from inverse box model of the region^26,27^. We note that one important difference between our approach and an inverse model is that estimates from inverse box model include production by entrainment that occurs under the mixed layer; a process excluded in our estimate that focuses on mixed-layer processes. Based on the calculation in the Weddell Sea, we deduce that only 1 Sv of water denser than 27.9*γ* subducts outside this region. We believe this value to be strongly underestimated due to the poor resolution of potentially key local and transient processes (e.g., coastal processes and polynyas) in our dataset. Important buoyancy fluxes are not likely to be captured within the very dense layers, especially in regions of intense polynyas activity such as the East Antarctica and the Ross Sea (See Supplementary Note 5 and Supplementary Fig. 6).

**Fig. 5:**
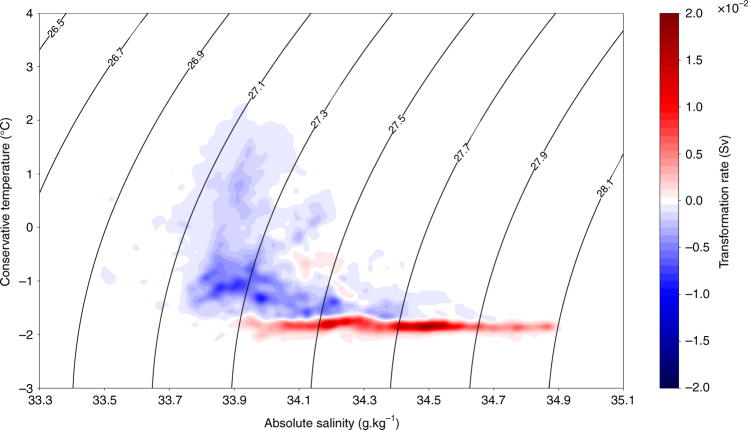
Annual-mean water-mass transformation rate in Sv in the T/S plane with neutral density contours superimposed. The positive values of the water-mass transformation rate indicate a transformation toward higher density classes (buoyancy loss), and the negative values indicate a transformation directed toward lighter density classes (buoyancy gain)

An alternative and complementary way to examine the variability of water-mass transformations within each density layer is to investigate transformation in temperature-salinity space, which allows us to distinguish some features that are overlooked when viewed only in neutral density space^28^. In temperature-salinity space (Fig. 5), water-mass transformations show a clear dipole, with a large-scale lightening of almost all the temperature-salinity bins of the surface ocean, and a densification confined in the coldest water-masses of the domain, close to the freezing point. Except for the lightest and densest layer of the domain, almost all densities are associated with water-masses that both lighten and densify, but the sign of the transformation is clearly separated by temperature: densification when the water is cold, mostly in fall and winter; and lightening when the water is warmer, mostly in spring and summer. In accordance with the transformation rates calculated in neutral density space, the transformations computed in temperature–salinity space indicate that waters around the characteristics 27.6*γ*/−1.5 °C/34.3 g kg^−1^ are associated with a divergence of transformation rate, associated with an upwelling from the deep ocean.

### Surface closure of the overturning circulation under sea-ice

Most of our current observation-based knowledge of the Southern Ocean meridional overturning circulation is founded upon large-scale inverse models^2,21,29^, but these models generally misrepresent polar processes such as sea-ice/ocean interaction, meaning that little is known about the near-surface closure of the overturning circulation south of the Antarctic Circumpolar Current. Consistent with our results, the most recently published global ocean inverse model estimated a southward transport of ~25 Sv of Circumpolar Deep Waters (e.g., 25.5 ± 5 Sv in ref. ^28^; ~ 20 Sv in ref. ^21^) in the density range ~27.3–27.9*γ*, and a northward return flow within lighter and denser density classes. In addition, we find that the estimates of water-mass transformation in the surface layer agree well with a complex assimilated solution^12^, both in terms of the density range and rate of overturning (though we do observe notable differences when disentangling freshwater and heat fluxes contributions; See Supplementary Note 3 and Supplementary Fig. 3). The overall agreement over a range of very different methods and approaches is to be noted and provides confidence in our understanding of the large-scale overturning of the Southern Ocean. The Southern Ocean overturning can be interpreted as a two-cell system: one upper cell associated with upwelling of mid-depth waters and their transformation into lighter waters; and one bottom cell associated with upwelling of mid-depth waters and their transformation into denser waters (Fig. 1). But this two-cell system can also be thought as a single connected pathway in a three-dimensional view^30^. In such a conceptual view of the overturning, dense CDW (Lower Circumpolar Deep Waters, LCDW) originating from the Atlantic basin are transformed in the polar Southern Ocean region into bottom waters (bottom cell), which are exported northward as Antarctic Bottom Waters in the Pacific and Indian basins, where they lighten into CDW (Upper Circumpolar Deep Waters, UCDW), before re-entering the Southern Ocean, and being converted into lighter waters (upper cell). In the three-dimensional framework, LCDW of typical density ~28*γ* is upwelled and converted into denser waters, and UCDW of typical density ~27.7–27.8*γ* is upwelled and converted into lighter waters. In order for the surface fluxes inferred from this study to match this scenario, LCDW and UCDW need to be converted to lighter water before they reach the surface, so LCDW can reach the surface ocean in a density class lighter than 27.9*γ* (which are converted into denser water at the surface), and UCDW can reach the surface in a density class lighter than 27.6 (which is converted into denser water at the surface). Diapycnal mixing beneath the mixed-layer is responsible for the conversion before water enters the mixed-layer. This view is consistent with the model estimate of Abernathey et al.^12^, who found that almost ~10 Sv of water is transformed to lighter density class by diapycnal mixing at all densities across UCDW and LCDW in the upper 700 m of the ocean. Upper-ocean transformation of water-masses within the ocean interior before they reach the mixed-layer is likely be an important component of the overturning circulation that needs to be clarified in future work.

Previous work has mostly discussed the ocean circulation response to cryosphere change in terms of weakening convection due to increased stratification^31–33^. In the present study, we show that sea-ice change, or even change in the regional distribution of sea-ice, as observed during the last few decades^34^, could have a major role in modifying surface water-mass transformation and the overturning circulation, with critical implication for the global carbon cycle^35–37^. Present climate models vary widely in their ability to represent sea-ice and the Southern Ocean freshwater cycle^38^, and thus their present and future rates of overturning circulation and associated carbon cycle^39^. Our results provide a novel observationally-based estimate of the exchanges between the mixed-layer and the ocean interior in the sea-ice sector that can be used to assess the ability of models to represent the closure of the meridional overturning circulation in this key region. Additionally, our calculations of the large-scale overturning circulation are based on ongoing long-term funded international observation programs. Thus, the method described here could be used in the future to diagnose variability in circulation rates in a climate change context.

## Methods

### Water-mass transformation and formation framework

In this paper, we compute water-mass transformation and formation based on the study by Tziperman and Speer^20^, which used a framework first introduced by Walin^16^. Water-mass transformation is defined as the volume flux of a water-mass consumed or produced by buoyancy forcing in a given density class. In contrast, water-mass formation refers to the convergence or divergence of transformed water-masses, and can be associated with subduction and upwelling through the base of the ocean mixed-layer. Buoyancy forcing considered here can be either surface buoyancy fluxes (exchange of buoyancy between the ocean and atmosphere or between the ocean and cryosphere), or diapycnal mixing in the ocean interior.

The surface buoyancy flux ${\cal B}(x,y,t)$ can be expressed as a function of location and time as follows:

1 $${\cal B}(x,y,t) = - \frac{g}{\rho }\left[ {\frac{{\alpha {\kern 1pt} H}}{{C_p}} + \beta ({\mathrm{FWF}} \cdot S)} \right]$$

where *α* is the thermal expansion coefficient at constant pressure defined by *α* = −(1/*ρ*) × ∂*ρ*/∂*T*, *β* is the saline contraction coefficient at constant pressure defined as *β* = (1/*ρ*) × ∂*ρ*/∂*S*, *C*_*p*_ is the specific heat of seawater, *ρ* is the density of seawater, *H* represents the surface heat flux (in W m^−2^), FWF is the surface freshwater flux from evaporation, precipitation and ice formation/melt (in m s^−1^), and *S* is the mean salinity in the mixed-layer. Here, we express the heat flux (first term in the right hand side of Eq. (1)) and the freshwater flux (second term in the right hand side of Eq. (1)) as equivalent heat fluxes in W m^−2^, for easier comparison of their respective contributions. The convention used in this study is that a negative flux corresponds to a buoyancy flux out of the ocean, i.e., the ocean surface layer loosing buoyancy and becoming denser (e.g., cooling, evaporation and brine rejection).

In addition to surface buoyancy fluxes, diapycnal fluxes can arise from mixing (either vertically through the base of the mixed-layer, or horizontally through outcropped isopycnal surfaces), which we will refer to in the study as ${\cal R}(x,y,t)$.

The annual-mean transformation rate *F*(*σ*_0_), expressed in *m*^3^.*s*^−1^, in a given potential density class *σ*_0_, associated with a diapycnal flux, ${\cal D} = {\cal B} + {\cal R}$, corresponds to the yearly integrated contribution of diapycnal fluxes to the density class:

2 $$F\left( {\sigma _0} \right) = {\int}_{\mathrm{year}} {\kern 1pt} \mathrm{d}t{\int} {\int}_{\mathrm{area}} {\kern 1pt} \mathrm{d}x\mathrm{d}y{\kern 1pt} {\cal D}(x,y,t)\delta \left( {\sigma (x,y,t) - \sigma _0} \right),$$

where *δ* is a delta function equal to zero except when mixed-layer density is within the range $\left[ {\sigma _0 - \frac{1}{2}\Delta \sigma } \right.$ : $\left. {\sigma _0 + \frac{1}{2}\Delta \sigma } \right]$.

The water-mass formation, *M*(*σ*)^24,40,41^, is defined as the water that accumulates over 1 year between two successive isopycnals, *σ*_1_ < *σ* < *σ*_2_

3 $$M(\sigma ) = - \left[ {F\left( {\sigma _2} \right) - F\left( {\sigma _1} \right)} \right]$$

Since we are working on upper-ocean processes, we compute the transformation/formation rates with respect to surface-referenced potential density as introduced in the above prognostic equations. Then, in order to identify the well-known water-masses in neutral density coordinates, we interpolate before each plot the potential density to neutral density (see Supplementary Note 4 and Supplementary Fig. 5). We use the relationship $\gamma \simeq \gamma (\sigma )$, presented in Supplementary Note 4, to convert all results from potential density to neutral density. All figures in this study are presented in neutral density coordinates.

### Computation of water-mass transformation and formation

As shown in Eq. (2), two critical pieces of information are needed to compute annual-mean water-mass transformation and formation. The first of these is the climatological seasonal cycle of potential density (*σ*) in the ocean surface mixed layer. The second is the climatological seasonal cycle of the diabatic flux, ${\cal D}$, in the ocean surface mixed layer. In this paper we infer these two terms using a large database of hydrographic observations in the Southern Ocean sea-ice sector. We define the sea-ice sector as the region seasonally capped by sea-ice, i.e., the region south of the winter (September) sea-ice extension with an ice concentration greater than 15%. The seasonal cycle of sea-ice extent is estimated using a climatological-mean (2000–2015) satellite-derived observations from Nimbus-7 SMMR and DMSP SSMI/SSMIS passive microwave data at 25 km resolution.

The international Argo project revolutionized our knowledge of the oceans over its entire seasonal cycle and in the middle of the basins, far from repeat hydrography lines and coastal regions. In particular, in the historically poorly sampled Southern Ocean, the Argo project provided a detailed understanding of the mixed-layer and its density^42,43^. However, until recently, Argo floats were unable to sample in the sea-ice sector. In combination with the Argo program, and traditional ship based observations, Pellichero et al.^13^ used observations from instrumented marine mammals^44^ that widely cover the Southern Ocean sea-ice sector over its entire seasonal cycle. These complementary databases produced a robust climatology of Southern Ocean mixed-layer characteristics in the sea-ice sector, which we use in this study to compute an observation-based seasonal cycle of potential and neutral density in the Southern Ocean mixed-layer. As such the water-mass transformation calculations are applied to the Southern Ocean mixed-layer which varies from tens of meters in summer to more than 200 m during winter (see Pellichero et al.^13^ for more details).

Surface diapycnal fluxes are composed of ocean buoyancy exchanges with the atmosphere and the cryosphere, as well as diapycnal mixing (both vertical and horizontal). Ocean buoyancy fluxes are commonly obtained from atmospheric reanalysis datasets. Unfortunately, in the Southern Ocean such reanalysis products are poorly constrained, and essentially unusable in the seasonally sea-ice covered region. Instead, we derive our own unique estimate of surface diapycnal fluxes product based on the climatological seasonal cycle of ocean surface layer characteristics under sea-ice^13^.

The mixed-layer heat and freshwater budget can be expressed as

4 $$\frac{{S_{\mathrm m}}}{{h_{\mathrm m}}} \cdot {\mathrm{FWF}} + {\cal R}_{\rm S} = \frac{{\partial S_{\rm m}}}{{\partial t}} + u_{\rm e} \cdot \nabla S_{\rm m} + u_{\rm g}.\nabla S_{\rm m} + \frac{{w_{\rm e}{\mathrm{\Delta }}S_{\rm m}}}{{h_{\rm m}}},$$

and

5 $$\frac{H}{{\rho _0C_ph_{\rm m}}} + {\cal R}_{\rm T} = \frac{{\partial T_{\rm m}}}{{\partial t}} + u_{\rm e}.\nabla T_{\rm m} + u_{\rm g}.\nabla T_{\rm m} + \frac{{w_{\rm e}{\mathrm{\Delta }}T_{\rm m}}}{{h_{\rm m}}},$$

where *u*_e_ is Ekman velocity, *u*_g_ is geostrophic velocity, *S*_m_ and *T*_m_ are respectively the mixed-layer absolute salinity and conservative temperature, *h*_m_ is the mixed-layer depth, *w*_e_ is the entrainment velocity, and ${\cal R}_{\rm S}$ and ${\cal R}_{\rm T}$ are respectively salinity and temperature diapycnal mixing in the ocean mixed layer (both lateral and vertical).

Each of the terms on the right hand side of Eqs. (4) and (5) are computed as in Pellichero et al.^13^. In addition, we compute the lateral geostrophic advection term from the mean dynamic topography (MDT) provided by AVISO for the period 1993–2012 (http://www.aviso.altimetry.fr/). Using a range of estimates of FWF and *H*, Pellichero et al.^13^ showed that the budgets are relatively well closed in the sea-ice sector. In this paper, instead of using one of the existing products for FWF and *H* in the sea-ice sector, we have derived our own estimates of $\mathrm{FWF} + {\cal R}_S$ and $H + {\cal R}_T$ from Eqs. (4) and (5). The existing products for FWF and *H*, are instead used for comparative purpose, and help place an error bound estimate on our calculations (See Supplementary Note 3 and Supplementary Fig. 4). We consider four products: two of which have been produced by Tamura et al.^45^ by using the reanalysis products NCEP2 and ERA, but coupled with sea-ice observations, and we hereafter refer to them as “Tamura (NCEP2)” and “Tamura (ERA)”; a third product has been developed by Petty et al.^46^ and is based on a mixed-layer model incorporated into a sea ice model CICE and we hereafter refer to it as “Petty (CICE)”; finally a fourth product is an output of an ocean model (NEMO3.5) coupled to a sea-ice model (LIM3.6) which has been produced by Barthélemy et al.^47^, which we hereafter refer to as “Barthélemy (NEMO).” Each of these products have their own limitations and constraints, but taken together, give a sense of where our observation-based estimate stands when compared to those that are state of the art.

### Error estimate and propagation

Water-mass transformation (Eq. (2)) is computed from the knowledge of buoyancy fluxes in the mixed layer (${\cal D}(x,y,t)$), and the mixed-layer density (*σ*(*x*, *y*, *t*)). Below, we detail how we compute the error on buoyancy fluxes, and error on the mixed-layer density field, before describing how each of those errors are propagated in the computation of water-mass transformation.

Error from surface buoyancy flux ($\varepsilon _{\cal D}$): As stated in the previous section, surface buoyancy fluxes are computed from Eqs. (4) and (5). Errors on the computation of surface buoyancy fluxes arise from the mixed-layer detection method, the instrumental errors in measurements of pressure, temperature, and conductivity and the spatial sampling. All of these errors are presented in Pellichero et al.^13^ and we use them to compute errors pertaining to each of the terms on the right hand side of Eqs. (4) and (5). The total error for the surface buoyancy fluxes is computed for each month of the year, and taken as the sum of errors arising from each term on the right hand side of Eqs. (4) and (5). The annual-mean error fields are presented in Supplementary Fig 6. Largest errors are found co-located with flux maxima and are greater for the freshwater flux than the heat flux.

Error from mixed-layer density (*ε*_*σ*_): The error of the mixed-layer density field is computed from the errors associated with mixed-layer salinity and temperature presented in Pellichero et al.^13^. Error estimates of temperature and salinity include instrumental error, error from the mixed-layer depth detection method, and optimal interpolation errors.

Propagation of the error: The water-mass transformation (Eq. (2)) is computed by discretizing the integral as a sum. Equation (2) can be rewritten

6 $$F = \mathop {\sum}\limits_i^n \mathop {\sum}\limits_j^n \mathop {\sum}\limits_t^n C\left[ {{\cal D}_{i,j,t}, \ldots ,\sigma _{i,j,t}, \ldots } \right]$$

where *C* = ${\cal A}_{i,j} \times {\cal D}_{i,j,t} \times \delta \left( {\sigma _{i,j,t}} \right)$, and ${\cal A}_{i,j}$ is the area of the corresponding longitude × latitude grid bin (*i*,*j*) at time *t*, and ${\cal D}_{i,j,t}$ and *σ*_*i*,*j*,*t*_ are the surface buoyancy fluxes and surface density at the longitude × latitude grid bin (*i*,*j*) at time *t*.

From error propagation theory, one can write the covariance of F in terms of covariance of ${\cal D}_{i,j,t}$ and *σ*_*i*,*j*,*t*_ (${\cal A}_{i,j}$ has no associated error):

7 $${\rm cov}(F) = J{\kern 1pt} {\rm cov}(C)J^T$$

with:

Assuming that errors in surface buoyancy fluxes and density have no spatial and temporal correlation, one can write: ${\rm cov}\left( {{\cal D}_{i,j,t}} \right)$ = ${\rm Diag}\left( {\varepsilon _{{\cal D}_{i,j,t}}^2} \right)$, and ${\rm cov}\left( {\sigma _{i,j,t}} \right)$ = ${\rm Diag}\left( {\varepsilon _{\sigma _{i,j,t}}^2} \right)$, where Diag is a diagonal matrix.

The terms of the matrix J can be expressed as:

8 $$\frac{{\partial F}}{{\partial {\cal D}_{i,j,t}}} = {\cal A}_{i,j} \times \delta \left( {\sigma _{i,j,t}} \right)$$

9 $$\frac{{\partial F}}{{\partial \sigma _{i,j,t}}} = {\cal A}_{i,j} \times {\cal D}_{i,j,t} \times \frac{{\partial \delta \left( {\sigma _{i,j,t}} \right)}}{{\partial \sigma _{i,j,t}}}$$

The derivative of a delta function is not defined where its argument is zero. However, the delta function can be approximated by a normal distribution with a small standard deviation:

$$\delta \sigma _{i,j,t} = e^{ - \frac{{\left( {\sigma - \sigma _{i,j,t}} \right)^{10}}}{{2a^{10}}}},{\mathrm{with}}{\kern 1pt} {{a}} = {\mathrm{0}}{\mathrm{.05}},$$

which provides a derivative of the delta function for the purpose of error propagation.

By combinging these terms in Eq. (7), we find that the error on F, expressed as its standard deviation, std(F), is:

10 $${\rm std}(F) = \sqrt {{\rm cov}(F)} ,$$

with:

11 $$\begin{array}{*{20}{l}} {{\rm cov}(F)} \hfill & = \hfill & {\mathop {\sum}\limits_{i,j,t}^n \left[ {{\cal A}_{i,j} \times \varepsilon _{{\cal D}_{i,j,t}} \times \delta \left( {\sigma _{i,j,t}} \right)} \right]^2} \hfill \\ {} \hfill & {} \hfill & { + \mathop {\sum}\limits_{i,j,t}^n \left[ {{\cal A}_{i,j} \times {\cal D}_{i,j,t} \times \varepsilon _{\sigma _{i,j,t}} \times \delta \left( {\sigma _{i,j,t}} \right)} \right]^2} \hfill \\ {} \hfill & {} \hfill & { \times \left( {\frac{{5\left( {\sigma - \sigma _{i,j,t}} \right)^9}}{{a^{10}}}} \right)^2} \hfill \end{array}$$

The first term on the right hand side of the Eq. (11) corresponds to the source of error coming from errors in the surface buoyancy flux (Supplementary Fig. 7c), while the second term corresponds to the source of error coming from errors in the density field (Supplementary Fig. 7b). The major source of error in the calculation of water-mass transformation originates from surface buoyancy fluxes, whereas the density-based error contribution is minor (Supplementary Fig. 7).

### Data availability

All relevant data are available from the authors.

## Electronic supplementary material

Supplementary Information
