# Supplementary material for: The southern ocean meridional overturning in the sea-ice sector is driven by freshwater fluxes
Source: Nat Commun. 2018 May 3;9:1789. doi: 10.1038/s41467-018-04101-2 (PMC5934442; doi:10.1038/s41467-018-04101-2)
Supplement: Supplementary file 1 — Supplementary Information [file 41467_2018_4101_MOESM1_ESM.pdf]

Supplementary Information:

The Southern Ocean Meridional Overturning In The Sea-Ice Sector is driven by freshwater fluxes

Pellichero et al.

Supplementary Information

Accompanying the article "The Southern Ocean Meridional Overturning in the sea-ice sector is driven by freshwater fluxes" by Pellichero, V., Sallée, J-B., Chapman, C. C. and Downes, S. M.

In this supplementary Information section, we present several figures and additional information in support of the main article text.

Supplementary Note 1: Seasonal cycle of water-masses transformation

Supplementary Fig. 1 illustrates the seasonally-averaged water-mass transformation rate. The largest rate of water-mass transformation occurs in summer (Dec-Jan-Feb), and all of this summer transformation is directed towards lighter density classes (with a peak at 27.3γ reaching about 22.5 Sv). This signal is consistent with the impact of sea-ice injecting large amounts of freshwater into the mixed-layer in summer as the sea ice is melting, as well as possible additional freshening by precipitation. Similarly, in spring (Sep-Oct-Nov) we observe a buoyancy gain, but with a slightly smaller magnitude and shifted to higher density classes: the peak of transformation reaches $\sim$10 Sv at 27.5γ. In contrast with spring and summer, buoyancy loss is observed at all densities in fall (Mar-Apr-May) and winter (Jun-Jul-Aug). In winter, the transformation rate is larger, reaching 12 Sv at 27.5γ, then drecreasing progressively until 28.8γ. This winter transformation is consistent with sea-ice releasing salty brine into the mixed-layer as the ice is forming.

Supplementary Note 2: Decomposition of the freshwater buoyancy flux

Supplementary Fig. 2a shows the water-mass transformation from our calculation (black line is the total WMT, and blue line is the freshwater contribution), decomposed into several contributions. Insofar as heat fluxes show a small contribution to the total buoyancy fluxes in the sea-ice zone, we explore only the contribution of surface buoyancy freshwater flux. To do so, we analyze different products that estimate the different contributions to the freshwater flux at the ocean surface: a surface freshwater flux product from the formation/destruction of sea-ice and the lateral transport of sea-ice across the surface[1], a freshwater flux product from the icebergs melting[2], and three freshwater flux products from precipitation/evaporation (CFSR, JRA55 and NCEP). We use the three precipitation/evaporation products in the same framework as presented in this study and estimate of the error on this term by the taking the standard deviation of all three products in each neutral density bin (red shaded area in Supplementary Fig. 2a. We note however that the estimate of the transformation by precipitation has very important limitations. First, the precipitation flux is very poorly known in the Southern Ocean, and is difficult to estimate from observations. Indeed, when precipitation falls on sea-ice, the freshwater associated with precipitation is deposited onto ice floes themselves, and affects the ocean mixed-layer only when sea-ice melts or breaks apart, often likely in a different density class to that within which it was deposited. Taking account of such sea-ice drift would be both too complex and inaccurate for our purposes. Instead, we make the strong and almost certainly incorrect assumption that precipitation affects ocean mixed-layer in the same way as if there were no sea-ice at the location and time that the precipitation fell. Here, we make no claim of accuracy regarding the details of the water-mass transformation: we simply attempt to evaluate first order relative order of magnitude. Despite such important limitation, we find the sum of all freshwater components (in green Supplementary Fig. 2b) matches

relatively well with our estimated freshwater flux contribution to water-mass transformation. With all the care needed, this calculation suggests that precipitation and sea-ice freshwater fluxes contribute approximately to equal amount to the lightening of the mixed-layer, while the densification is mostly associated with sea-ice fluxes. Freshwater flux from iceberg melt is found negligible in the net water-mass transformation (though could be important locally in setting mixed-layer characteristics).

Supplementary Note 3: Comparison of water-mass transformation rates computed with alternative estimates of buoyancy fluxes and with previous studies

While the Methods section of the main text details our error estimate on water-mass transformation through a formal error propagation, an alternative way to assess errors on water-mass transformation is to repeat our calculations with a range of existing buoyancy flux products. We use freshwater and heat fluxes from four products: two coming from reanalysis-based estimates published by Tamura et al.[3], and two from numerical models of sea-ice coupled with an mixed-layer model[4], or with a realistic ocean[5]. Despite the wide differences in the methods used to produce the four buoyancy fluxes, their resulting water-mass transformation rates are remarkably similar to one another (Supplementary Fig. 4). Overall, all of the buoyancy flux products used in this study agree that lighter components of deep waters are generally transformed into lighter waters; and heavier waters are generally transformed into heavier waters, with a comparable rate of transformation. The rate of water-mass transformation into lighter densities are in good agreement, with a larger spread across the products in water-mass transformation rates for denser waters. The standard deviation of all these results can also be used as an alternative measure of error estimate of our calculation and is presented in Supplementary Fig. 4b. However, one limitation of this measure of error is that all of these fluxes are applied to the same observed mixed-layer density field. To release such constraint, we also compare below our results with an entirely independent study that performed a similar calculation to those described in this study, but using the output of a complex, data assimilating numerical model, the Southern Ocean State Estimate (SOSE)[6].

Supplementary Fig. 3 shows a comparison between the present study's estimate of water-mass transformation rate from heat and freshwater fluxes (thick lines), and the estimate published by Abernathey et al., 2016[6] (dashed lines). One main difference between our study and Abernathey et al.'s study, is that we focus our study in the sea-ice sector only, while Abernathey et al., 2016[6] produced an estimate for the entire Southern Ocean (south of 30°S). Therefore, in Supplementary Fig. 3, we gray shaded density classes that, in our observation-based product, are never found in the sea-ice sector, and density classes that are partly found in the sea-ice sector (dashed area), and density bins that are entirely found under sea-ice (white area). The comparison between the two WMT estimates is only possible in the sea-ice sector (white area). Indeed, in other density bins, large areas of the density class not found in the sea-ice zone, so additional fluxes might be accounted for in Abernathey et al., 2016[6] which are not accounted for in our study. In the sea-ice sector (white area), the total transformation fluxes compare remarkably well. However, we note some discrepancies when looking at the decomposition into heat and freshwater components. Despite such differences, the agreement between two very different approaches gives confidence in the robustness of the general results.

Supplementary Note 4: Relation between sigma and gamma

As stated in the Methods section, we computed the water-mass transformation calculations with respect to surface-referenced potential density (σ), and then the results are converted from sigma into neutral density (γ), for easier comparison with previous studies[7–9]. This conversion is done in the same way as in Abernathey et al., 2016[6], using a linear relationship between σ and γ.

We quantified this linear relationship from observation-based climatology of the Southern Ocean mixed-layer published in Pellichero et al., 2017[10]. The relationship is shown in Supplementary Fig. 5, and is strongly consistent with the one used in Abernathey et al., 2016[6].

Supplementary Note 5: Error estimate

As detailed in the Methods section, we compute the error in the buoyancy fluxes and the error in the mixed-layer density field, before including both in the computation of water-mass transformation. Here we present the result of the annual-mean error fields.

The Supplementary Fig. 6 highlights the error on our observation-based estimate of buoyancy flux (Supplementary Fig. 6c). The error mainly comes from the surface freshwater flux (Supplementary Fig. 6a) and its regional distribution is very localized at the ice edge where and along the coast of the Antarctic continent. By decomposing this freshwater signal (see Equation. 4), we find that most of this error emanates from the geostrophic advection term produced by AVISO (not shown).

The first term on the right hand side of the Equation. 11 corresponds to the error on the surface buoyancy flux (Supplementary Fig. 7c) whereas the second term represents the error on the surface density (Supplementary Fig. 7b). We combine both as explain in the Methods section in order to obtain the error field introduces in Supplementary Fig. 7a. The major source of error in the calculation of water-mass transformation comes from the surface buoyancy fluxes, whereas we find a minor contribution from the density bins.

Supplementary References

1. Haumann, F. A., Gruber, N., Münnich, M., Frenger, I. & Kern, S. Sea-ice transport driving southern ocean salinity and its recent trends. *Nat.* **537**, 89–92 (2016).

2. Merino, N. *et al.* Antarctic icebergs melt over the southern ocean: Climatology and impact on sea ice. *Ocean. Model.* **104**, 99–110 (2016).

3. Tamura, T. & Ohshima, K. I. Mapping of sea ice production in the arctic coastal polynyas. *J. Geophys. Res. Ocean.* **116** (2011). URL http://dx.doi.org/10.1029/2010JC006586. DOI 10.1029/2010JC006586.

4. Petty, A. A., Holland, P. R. & Feltham, D. L. Sea ice and the ocean mixed layer over the antarctic shelf seas. *The Cryosphere* **8**, 761–783 (2014).

5. Barthélemy, A., Fichefet, T. & Goosse, H. Spatial heterogeneity of ocean surface boundary conditions under sea ice. *Ocean. Model.* **102**, 82–98 (2016).

6. Abernathey, R. P. *et al.* Water-mass transformation by sea ice in the upper branch of the southern ocean overturning. *Nat. Geosci.* **9**, 596–601 (2016).

7. Jackett, D. R. & McDougall, T. J. A neutral density variable for the world's oceans. *J. Phys. Oceanogr.* **27**, 237–263 (1997).

8. Speer, K., Rintoul, S. R. & Sloyan, B. The diabatic deacon cell. *J. physical oceanography* **30**, 3212–3222 (2000).

9. Badin, G., Williams, R. G., Jing, Z. & Wu, L. Water mass transformations in the southern ocean diagnosed from observations: Contrasting effects of air–sea fluxes and diapycnal mixing. *J. Phys. Oceanogr.* **43**, 1472–1484 (2013).

10. Pellichero, V., Sallée, J.-B., Schmidtko, S., Roquet, F. & Charrassin, J.-B. The ocean mixed-layer under southern ocean sea-ice: Seasonal cycle and forcing. *J. Geophys. Res. Ocean.* 1608–1633 (2017).

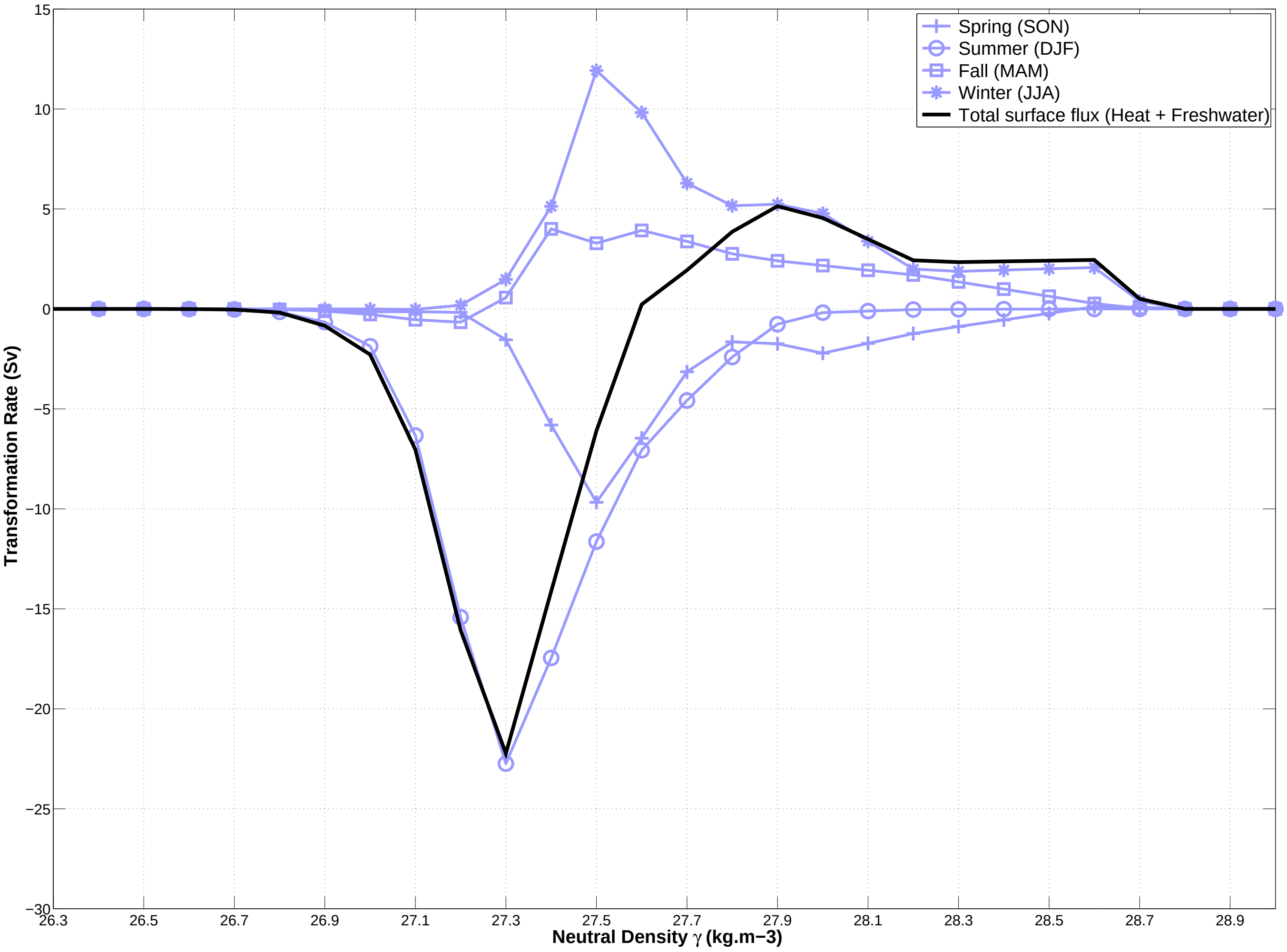

Supplementary Figure 1. Seasonal cycle of water-mass transformation under sea-ice. Annual-mean water-mass transformation (Sv) for the sea-ice sector in neutral density coordinates (black line) and the decomposition of the seasonal cycle into spring (SON, plus sign), summer (DJF, circle), fall (MAM, square) and winter (JJA, asterisk).

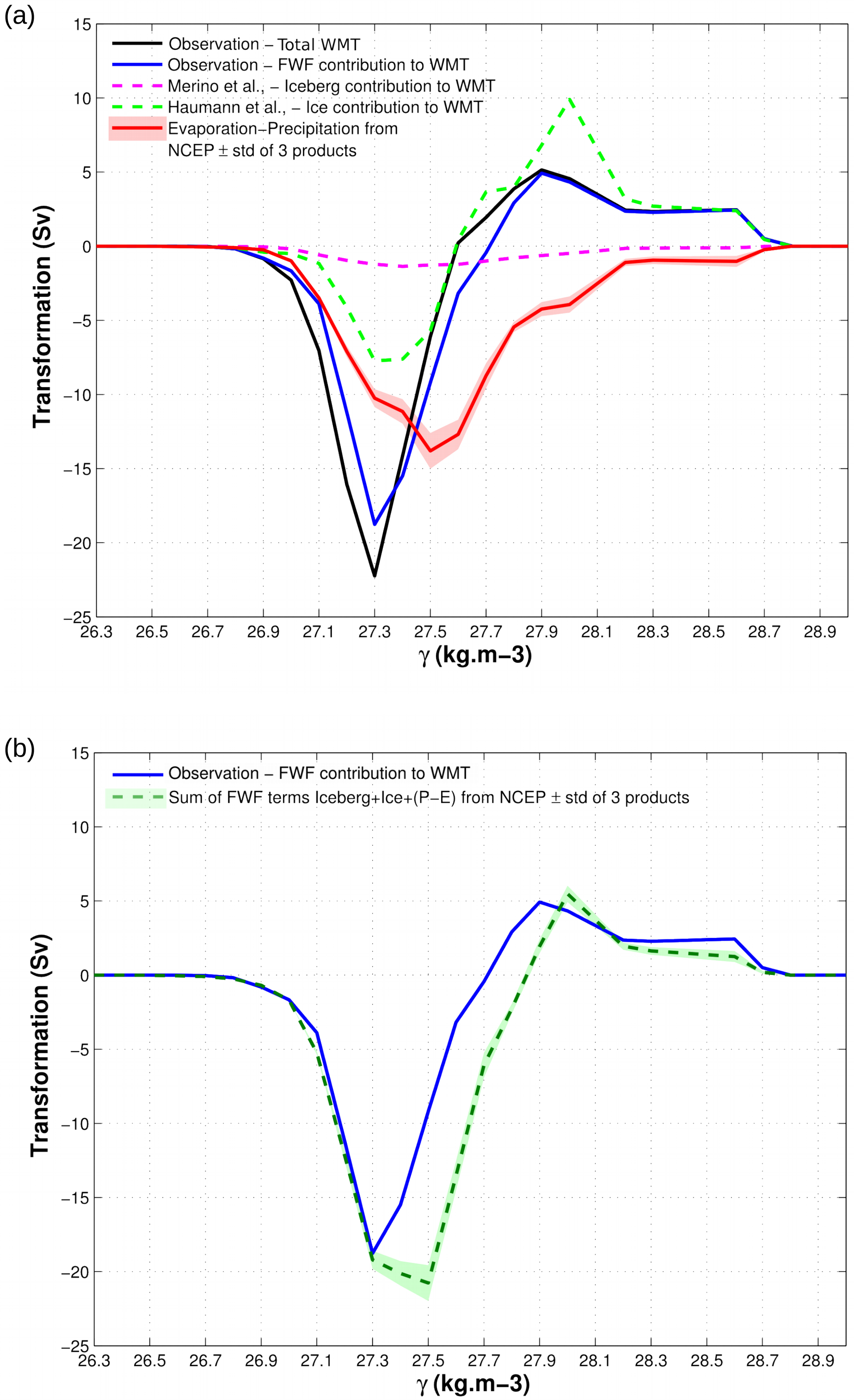

Supplementary Figure 2. Components of water-mass transformation in the sea-ice sector. (a) Decomposition of the freshwater flux responsible for the transformation of water-masses. The black line corresponds to our estimate of the net annual-mean transformation rate with the contribution of the freshwater flux in blue (see Fig. 3). The magenta line corresponds to the transformation induced by the freshwater from the icebergs melting. The green line is the transformation resulting from the sea ice growth/melt. Finally, the red line corresponds to the contribution to the freshwater flux from the precipitation/evaporation estimated from the NCEP product. The red shaded area is the standard deviation of 3 different precipitation/evaporation products, as an estimate of error on precipitation flux (CFSR, JRA55 and NCEP). (b) The dashed green line is the sum of all freshwater components i.e freshwater flux from sea-ice, precipitation (NCEP) and icebergs, and the shaded area represents estimated error from the precipitation flux (same as panel a)

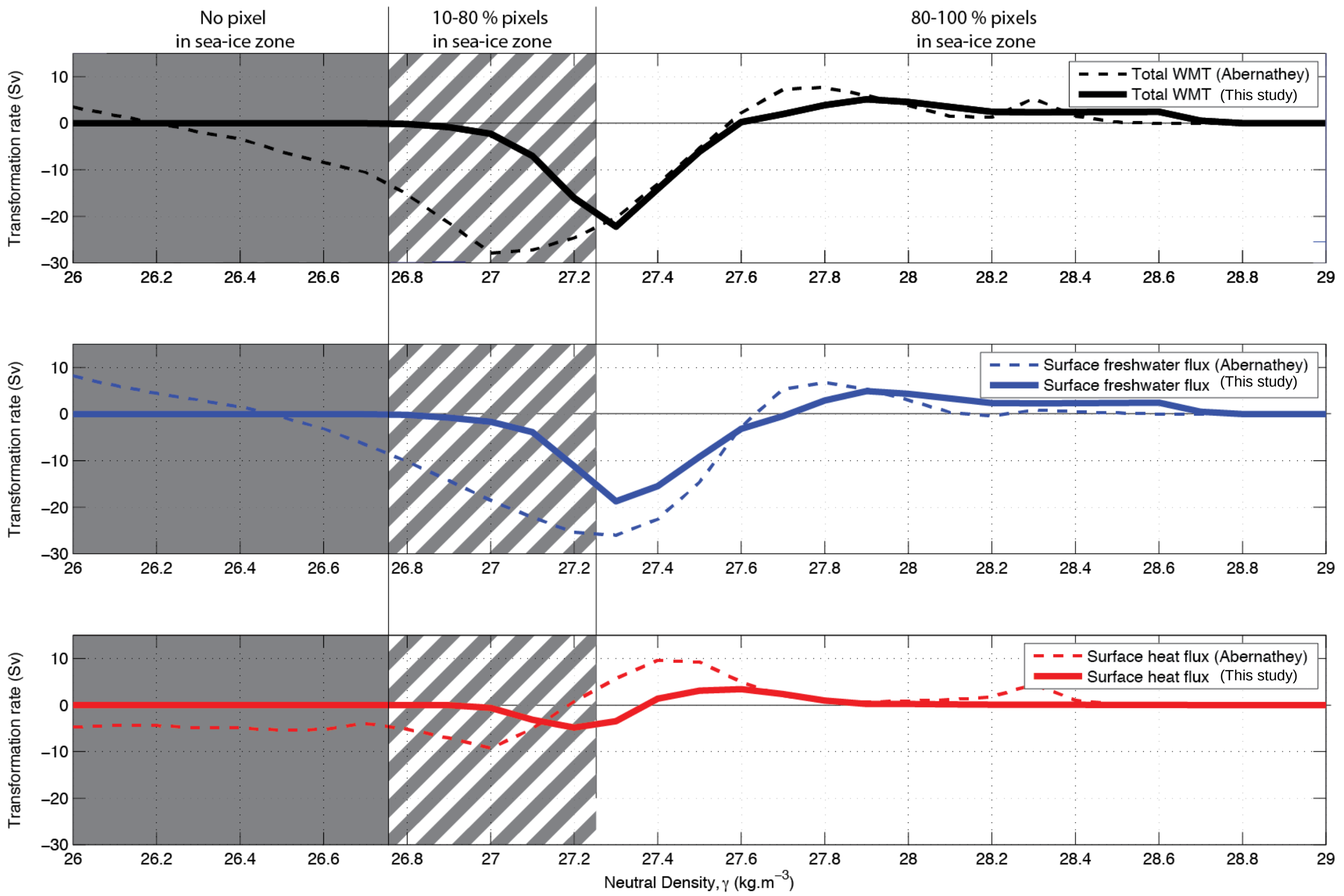

Supplementary Figure 3. Comparison of water-mass transformation rates between this study and Abernathey et al., 2016[6]. The upper panel shows our estimate of water-mass transformation rates (thick black line) compared to those of Abernathey et al., 2016[6] (dashed black line), and the decomposition into freshwater (middle panel) and heat (lower panel) fluxes. The gray area corresponds to the neutral density classes that outcrop outside the sea-ice zone; the striped area includes the density classes where 10-80% of the grid points are in the ice; and the white area is the density classes corresponding to the sea-ice sector where 80-100% of the grid points are under the sea-ice.

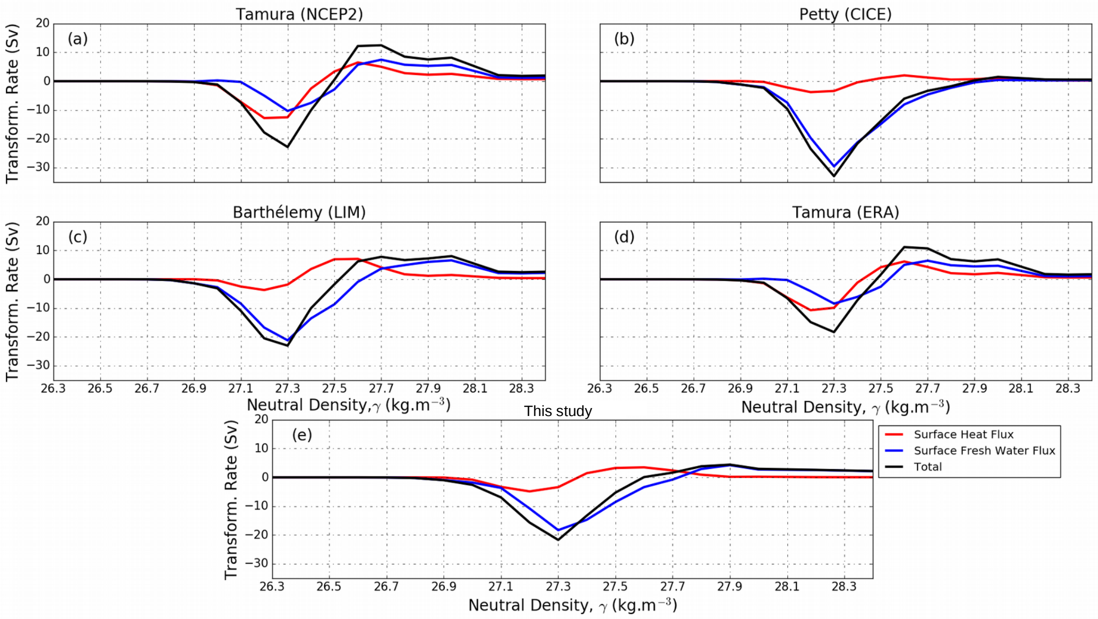

(f)

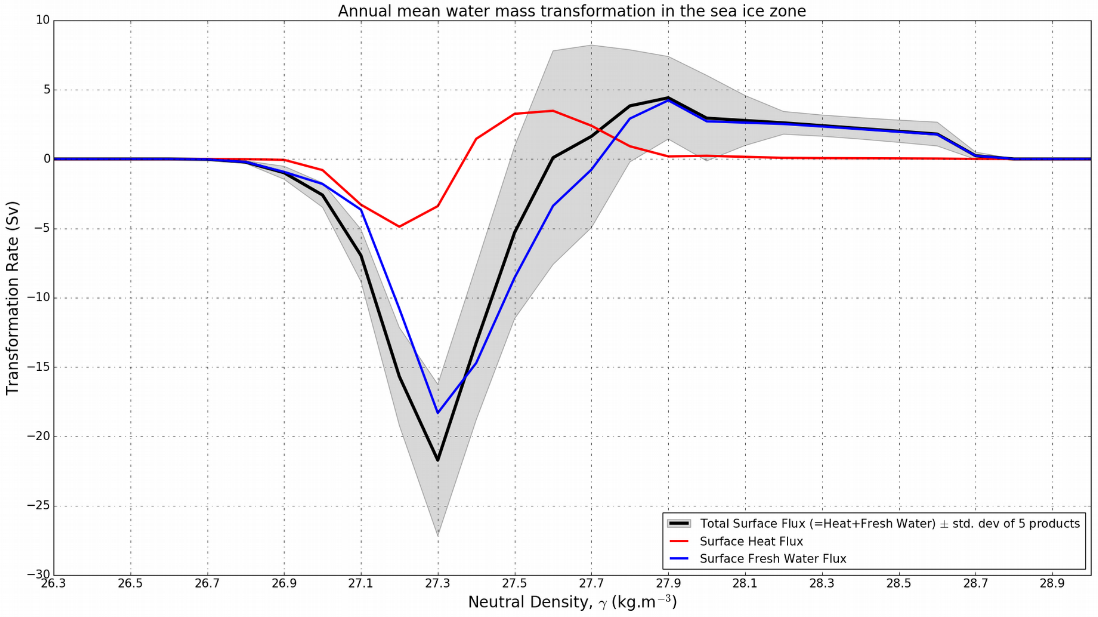

Supplementary Figure 4. Comparison between different estimations of water-mass transformation rate from existing buoyancy flux products. (a-e) Annual-mean water-mass transformation rate in the sea-ice sector from this study and 4 other surface buoyancy flux products. Each panel shows the water-mass transformation in the sea-ice sector (black line) with the surface air-ocean-ice freshwater flux contribution (blue dashed line) and the surface air-ocean-ice heat flux contribution (blue line) as following: (a) from Tamura (NCEP2), (b) from Petty (CICE), (c) from Barthélemy (LIM), (d) from Tamura (ERA) and (e) from this study. (f) Water-mass transformation rate with the error (gray shading) computed as the standard deviation of all these 5 products.

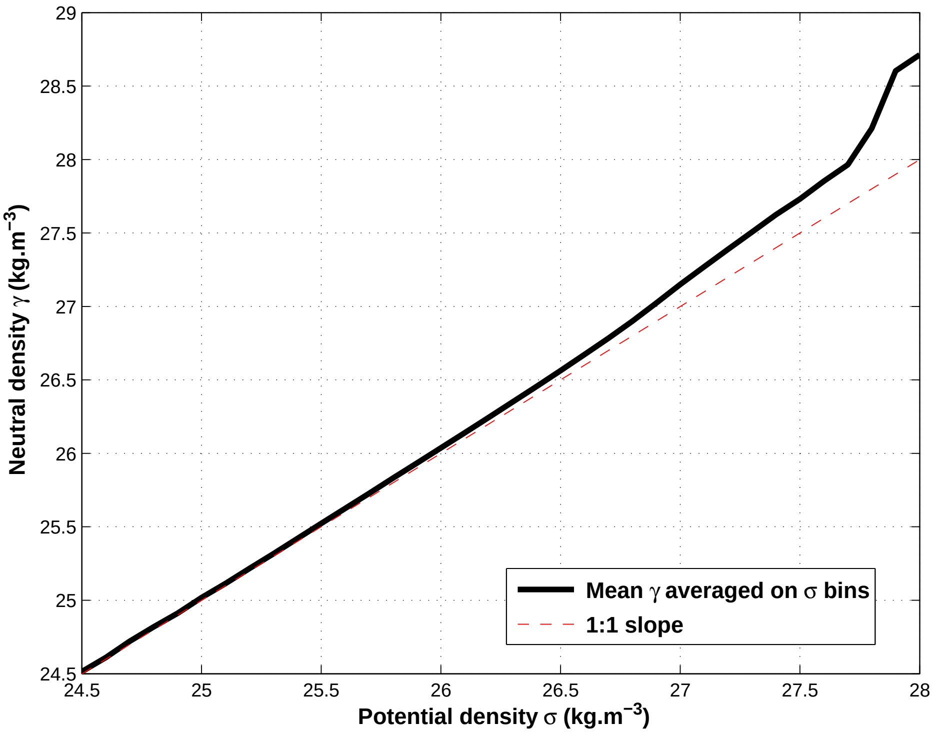

Supplementary Figure 5. **Relationship between potential density (σ) and neutral density (γ).** The black line presents the averaged neutral density in potential density bins of $0.05 kg.m^{-3}$. The red line is the linear function with 1:1 slope.

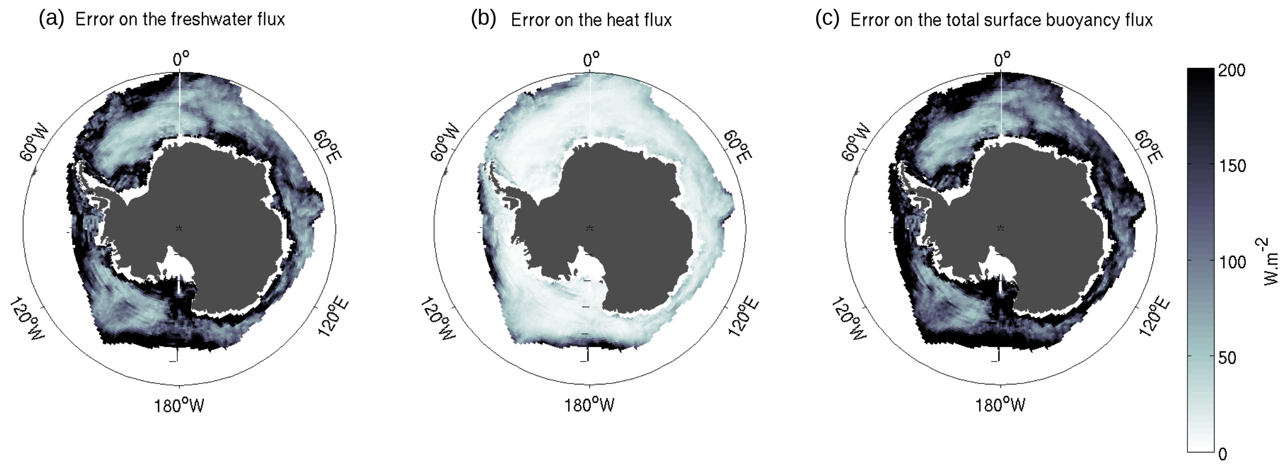

Supplementary Figure 6. Error in the atmosphere-ocean-ice flux in W.m^{-2} (c) decomposed into (a) the freshwater flux as estimated in Equation. 4 and (b) the heat flux as estimated in Equation. 5.

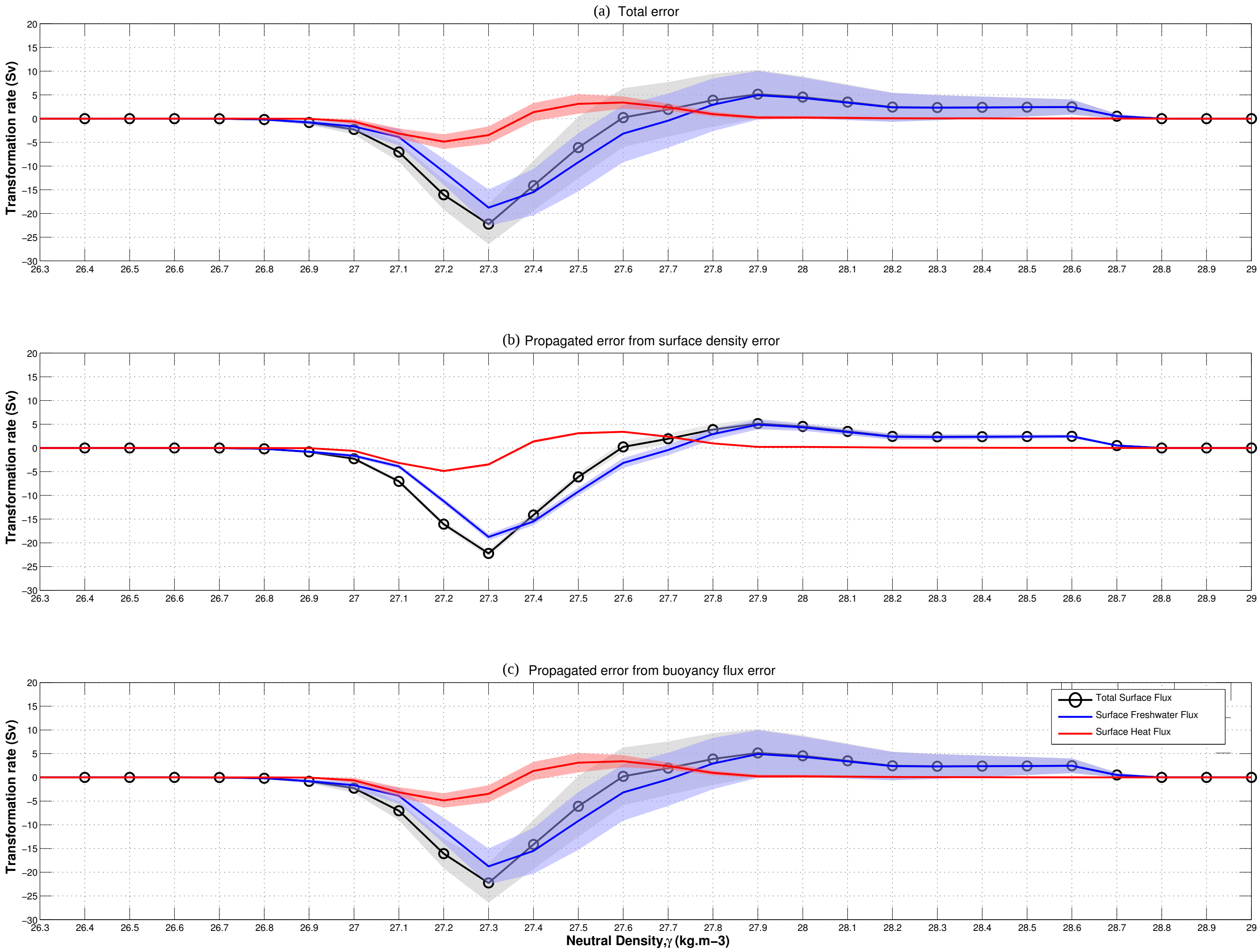

Supplementary Figure 7. Annual-mean water-mass transformation in Sv for the sea-ice sector (black line) decomposed into the freshwater component (blue) and the heat component (red). In all panels, shaded areas correspond to: (a) the total propagated error from both the buoyancy fluxes and the surface density error (see Equation. 10), (b) the source of errors in the density field, and (c) the source of errors in surface buoyancy fluxes.
